# Use of a hypercaloric and hyperprotein supplement enriched with immunonutrients in multi-pathological malnourished patients to improve the incidence and prevalence of pressure sores: A randomized controlled trial

**DOI:** 10.1371/journal.pone.0353933

**Published:** 2026-07-29

**Authors:** Eduardo Sánchez-Sánchez, Jara Díaz-Jiménez, Alejandro García-García, Alejandro Úbeda-Iglesias, Borja Muñoz-Tejada, Javier Martínez-Diegues, Cristina Sánchez-Fernández, Marina Erola-Moreno, Borja Vallecillo-Rico, Cristina Jiménez-Vázquez, Blanca Bohorquez-Almagro, Sol-Paula Cortés de Miguel, Antonio-Jesús Marín-Paz

**Affiliations:** 1 Department of Nursing and Physiotherapy, University of Cadiz, Cadiz, Spain; 2 Biomedical Research and Innovation Institute of Cadiz (INiBICA), Puerta del Mar University Hospital, University of Cadiz, Cadiz, Spain; 3 Punta de Europa University Hospital, Algeciras, Spain; 4 Department of Physiology, University of Granada, Granada, Spain; 5 Critical Care Medicine Department, Punta de Europa University Hospital, Algeciras, Spain; 6 Internal Medicine Department, Punta de Europa University Hospital, Algeciras, Spain; 7 Pharmacy Department, Punta de Europa University Hospital, Algeciras, Spain; 8 University Research Institute for Sustainable Social Development (INDESS), University of Cadiz, Jerez de la Frontera, Spain; PLOS: Public Library of Science, UNITED KINGDOM OF GREAT BRITAIN AND NORTHERN IRELAND

## Abstract

**Background:**

Predisposing factors for the development of pressure-related injuries (PRIs) include the presence of chronic diseases, polypharmacy, immobility and malnutrition. Nutrients such as arginine play an essential role in wound healing.

**Methods and findings:**

A single-blind randomized controlled trial was carried out. Intervention group received a complete hyperproteic and hypercaloric oral nutritional supplement with a fiber mixture enriched in omega 3, L-arginine and nucleotides, providing vitamin C and Zinc, and the control group received a hyperproteic and hypercaloric oral nutritional supplement with fiber mixture.

**Results:**

Descriptive analysis indicated a lower frequency of PRIs at 15 and 30 days in the Atémpero^®^ group compared to the control group, although these differences did not reach statistical significance. However, both improved at the end of the study. In addition, the high risk decreased in both groups, with a higher decrease in the Atémpero^®^ group (72.5% vs 40.9%), as well as the categorization and parameters of the PUSH scale, with a reduction of the same in the Atémpero^®^ group.

**Conclusions:**

While statistical significance was not achieved, the observed trends in PUSH scale scores and clinical observations within the Atémpero^®^ group suggest areas for further investigation. These preliminary data highlight a potential avenue for the nutritional management of multi-pathological patients, establishing a solid foundation for future large-scale confirmatory trials to validate the efficacy of immunonutrient-enriched ONS in pressure injury care. **Trial registration.** ClinicalTrials.gov NCT06726486.

## Introduction

Pressure-related injuries (PRIs) are included in dependence-related skin injuries (DRSI), along with moisture, friction/rubbing, and mixed or combined injuries [1]. PRIs are an essential health and clinical safety problem [2]. They are considered adverse effects of healthcare and have negative consequences for the health status of the population, the quality of life and the healthcare system in economic terms [3]. The prevention of PRIs remains a key priority area for healthcare professionals and a patient safety issue [4]. According to data reported by the 5^th^ National Prevalence Study of PRIs in Spain, the crude prevalence of PRIs in hospitalized adults was 7.0% [5].

This type of injury is one of the most frequent events in hospitalized patients worldwide. They are associated with negative impacts on patients and healthcare systems, leading to increased risk of nosocomial infection, pain and disability, and prolonged hospitalization, resulting in increased morbidity, mortality and financial costs to healthcare systems [6,7]. Guest et al. estimated the cost of managing chronic wounds in the UK to be £3,000 million annually [8].

They are often highly prevalent in the frail adult population, especially in those with chronic diseases. Multimorbidity or comorbidity in this population negatively influences the occurrence of PRIs [9,10].

Clinical practice guidelines for preventing and treating PRIs recommend different strategies, among which is maintaining optimal nutritional status, especially in the at-risk population [11], since malnutrition is a risk factor for developing and worsening PRIs [12]. PRIs occur at bony prominences and where there is compression of the underlying skin or tissue with an external surface. Factors that increase the likelihood of these pressure ulcers are immobility, incontinence and, as discussed above, poor nutritional status. Therefore, new interventions should be created among which nutrition optimization appears [13].

Malnourished patients are more vulnerable to developing pressure injuries and infectious complications. Adequate energy intake and macro- and micronutrients can improve wound development and healing. Interventions that help prevent or treat pressure injuries reduce wound care costs and healthcare demand [14]. Optimal nutritional intervention improves the healing of PRIs [15]. Deficiencies of protein, calories, vitamins, and minerals are logically implicated as contributing to skin degradation. The importance of nutritional assessment for pressure ulcer prevention is evident, as reflected by its inclusion in several society guidelines and risk factor assessment tools [16].

The relationship between malnutrition and PRIs is twofold: the response to injury can increase the metabolic needs of the area of injury, with large amounts of protein being lost through wound exudate. Thus, energy and protein needs in patients with chronic wounds may increase as cells involved in wound healing require protein for their formation and activity [2]. Moreover, malnutrition influences the immune response, decreases fibroblast activity, delays angiogenesis and reduces collagen formation [5]. Therefore, the European Pressure Ulcer Advisory Panel (EPUAP) states that sufficient calories and protein should be provided, promoting adequate vitamin and mineral intake. Individuals receiving medical care who are malnourished or at risk of malnutrition should receive expert nutritional assessment and intervention according to local practice [17].

Nutritional care of the multi-pathological population includes various techniques such as dietary counselling, meal fortification, increasing between-meal intake, oral nutritional supplements (ONS), enteral nutrition (EN) and parenteral nutrition (PN). During hospitalization, in malnourished patients or those at risk of malnutrition, ONS should be provided in order to improve dietary intake and decrease the risk of complications, such as PRIs and readmissions (grade of recommendation: A) [18]. In a multicenter study conducted in 2019, it was obtained that the most frequently performed nutritional interventions in patients with PRIs were assistance during intake (50.7%), an adaptation of the meal to the preferences of each patient (40.8%) and the performance of nutritional screening (39.4%). In other words, only 4 out of 10 patients underwent nutritional screening, and only 8.5% were prescribed oral enteral nutrition through nutritional supplements to meet their nutritional requirements [19]. In another study, the authors observed that between 22% and 38% of patients consumed < 50% of the food provided in the main meals, which increases the risk of malnutrition and the onset or delayed healing of PRIs [20].

On the other hand, patients with PRIs present a decrease in plasma arginine concentrations [21]. Among the amino acids, arginine plays an essential role in wound healing. It is a precursor of nitric oxide and proline, essential for the inflammatory process and collagen synthesis [22]. Arginine also stimulates growth hormone production and secretion, as well as T-cell activation [23]. Arginine enhances healing and modulates inflammation and immune response [18–20]. Evidence showed that arginine-enriched enteral nutrition led to a significant improvement in the healing of PRIs [24]. In Spain, the National Group for the Study and Advice on Pressure Ulcers and Chronic Wounds (GNEAUPP) published Technical Document No. XII, which states that the consumption of supplements that provide vitamins A, C, E, zinc and arginine improves the prevention (high level of evidence) and healing of PRIs (moderate level of evidence) [1].

Therefore, our study aims to evaluate whether using a hypercaloric and hyperproteic enteral formula enriched with immunonutrients reduces the incidence and prevalence of PRIs, improving their prevention and healing in hospitalized malnourished multipathological patients.

## Materials and methods

This study was reported according to the Consolidated Standards of Reporting Trials (CONSORT) Statement [25] (S1 Text).

### Design study

A single-blind parallel Randomized Controlled Clinical Trial (RCT) was conducted, where the investigator in charge of data analysis did not know the group to which each participant belonged. Data were collected from January 24, 2022, to April 12, 2024. The study was retrospectively registered in Clinical Trials with the code NCT06726486 (URL: https://clinicaltrials.gov/study/NCT06726486). The Ethics Committee for Drug Research of the Province of Cadiz approved the study on June 9, 2021, with registration number 84.21. All participants were informed of the study’s aims. Written informed consent was obtained from all participants and/or their legal representatives. Data confidentiality was guaranteed in accordance with the Helsinki Declaration and Spain’s Data Protection Act (Organic Act 3/2018).

### Sample

The sample size was determined using the formula for finite populations, based on the initial screening of 955 patients assessed for eligibility. A baseline sample of 48 participants with a 95% confidence level and a precision of 6% was used to estimate PRI incidence. This initial result was subsequently adjusted to account for an anticipated attrition rate of 30%, a conservative estimate given the high complexity and frailty of this multipathological cohort. Using the adjustment formula N = n/(1 − R), the final required sample size was calculated to be 69 participants.

#### Randomization.

The random allocation sequence was generated using R software (version 4.2.0) employing a process of simple randomization. A table of random numbers, created via the random number generation function in R, was the primary tool. One member of the research team randomly selected a starting point and a direction of movement until an adequate sample size for this type of study was reached. The resulting numbers were interpreted to assign participants: Even numbers were assigned to the intervention group, and odd numbers were assigned to the control group.

To conceal the allocation sequence, the generation and assignment process was automated via R software, ensuring the sequence was unpredictable. The investigator responsible for data analysis was the only team member who had access to the group allocation key after data collection was complete and prior to analysis. The personnel who recruited participants and the two research team members who assessed patient eligibility did not have access to the random allocation sequence at any point. Allocation was revealed only after a participant met all eligibility criteria and was enrolled in the study.

The study employed single-blinding, meaning the investigator responsible for data analysis was blinded to the intervention group assignments. Participants and care providers (medical and nursing staff) could not be blinded due to the distinctive nature of the Oral Nutritional Supplements (ONS) and the need to monitor their administration. However, outcome assessors (personnel who measured dependent variables such as the Braden scale and PUSH scale, as well as anthropometric and nutritional variables) were blinded to the intervention assignment.

#### Intervention.

Two groups of patients with non-equivalent ratios were created. The intervention group received the arginine-enriched ONS called Atémpero^®^. This ONS is a complete hyperproteic and hypercaloric diet with a mixture of fibers enriched in omega 3, L-arginine and nucleotides, which provides vitamin C and Zinc. This diet is specific for malnourished patients who present some wounds. There were no risks during the administration of this formula, and it is within the guidelines for approaching malnourished patients with some wounds. The control group is a complete high-protein, high-calorie diet with mixed fiber, indicated for malnourished patients with stress hyperglycemia and diabetes with increased protein and energy requirements. There were no methodological changes during the process. Both ONSs are suitable for diabetic patients and have very similar compositions, differing mainly in their composition of arginine and immunonutrients.

### Participants

The study population consisted of all hospitalized patients followed by the Internal Medicine Department of a regional hospital who wished to participate in the study at the time of recruitment and who met the inclusion criteria: chronic patients with complex needs being; malnourished or at risk of malnutrition; moderate-high risk of developing Pressure Injuries (PIs) or presence of PIs. The exclusion criteria were limitations in oral intake (due to pathologies, health status, among others) or contraindications for the use of the oral route and digestive intolerance or allergy to any of the components of the products. Two members of the research team assessed patient eligibility.

### Variables

The dependent variable under study was the presence of PRI, the risk of developing PRIs using the Braden scale as a measuring instrument, and the categorization of PRIs and PRIs healing using the PUSH scale (Pressure Ulcer Scale for Healing). The Braden Scale was selected as the primary instrument for risk assessment due to its high sensitivity and established predictive validity in acute care settings for multi-pathological patients. Complementarily, the Pressure Ulcer Scale for Healing (PUSH) was employed to monitor wound progression, as it provides a multidimensional evaluation—incorporating surface area, exudate amount, and tissue type—that is more sensitive to early physiological changes in wound bed preparation than simple staging. The 15 and 30-day time points were strategically chosen based on the metabolic window required for immunonutrients (for example: L-arginine and Zinc) to modulate the inflammatory phase and initiate collagen synthesis, which typically manifests clinically within the first two to four weeks of intensive nutritional support in malnourished individuals [26].

We collected sociodemographic variables (sex and age), clinical variables (degree of dependence using the Barthel test, chronic diseases, and others), anthropometric variables (weight, height, tricipital skin fold, subscapular skin fold, calf circumference, arm circumference, BMI, and body composition through electrical bioimpedance (model (50khz) Akern BIA 101), nutritional variables (nutritional status through the Mini-Nutritional Assessment (MNA), Global Leadership Initiative on Malnutrition (GLIM) criteria, % dietary intake and adherence to the ONS) and mortality.

Data were collected within 24 hours of hospital admission, at 15 and 30 days. Once the trial began, there were no variations in the variables or data collection.

#### Study monitoring.

Due to the nature of the study interventions, which consist of oral nutritional supplements with a well-established safety profile and for which no significant risks have been evidenced during their administration in similar populations, an independent Data Safety Monitoring Committee (DSMC/DMC) was not deemed necessary, as the intervention comprised commercially available oral nutritional supplements with an established safety profile.

Safety and data integrity monitoring were performed internally by the research team, particularly by the study coordinator, who continuously reviewed adverse events and data quality. Furthermore, ongoing ethical oversight was ensured by the Ethics Committee for Drug Research of the Province of Cadiz.

#### Data management.

Data collection was performed using standardized forms, ensuring consistency and accuracy. All collected data were entered into a secure electronic database hosted on the hospital server. Data entry was performed by a research technician and verified by a second research technician and the study coordinator to minimize transcription errors and ensure accuracy.

To ensure data quality, regular data cleaning procedures were implemented, including completeness checks, consistency checks, range checks, and detection of outliers. Any discrepancies or illogical values were queried with the study site for clarification and correction, in accordance with pre-established data management plans.

Participant confidentiality was maintained throughout the study. All participants were assigned a unique study identification number, and personal identifying information was stored separately from the research data in hard copy format under lock and key, with restricted access. Data were pseudonymized to protect participant privacy during analysis.

All relevant data underlying the findings of this study are fully available within the manuscript and its Supporting Information files (S1 Text), and the dataset can also be found in a public repository (Figshare). The authors confirm there are no ethical or legal restrictions on the public sharing of the de-identified participant data.

### Data analysis

The data obtained from the variables are represented descriptively. Qualitative variables are shown by frequency and percentage, and quantitative variables by mean and standard deviation or median and interquartile range.

For comparisons, the Chi-square test was applied to qualitative variables if no more than 20% of the expected cell counts were less than 5; otherwise, Fisher’s exact test was used. In the case of quantitative variables, the Student’s t-test for independent samples was performed when the data distribution was normal, using the Mann-Whitney U test in the case of variables that did not have a normal distribution.

Regarding missing data, no imputation methods were used; all analyses were based on complete cases for each variable.

The level of significance or type I error to be used is 0.05. Given the multiple outcomes assessed, *p*-values were adjusted using the Benjamini-Hochberg procedure (False Discovery Rate) to control for multiple comparisons. For this purpose, we will use the R statistical program and SPSS 25 jointly and in a coordinated manner.

## Results

**Fig 1:**
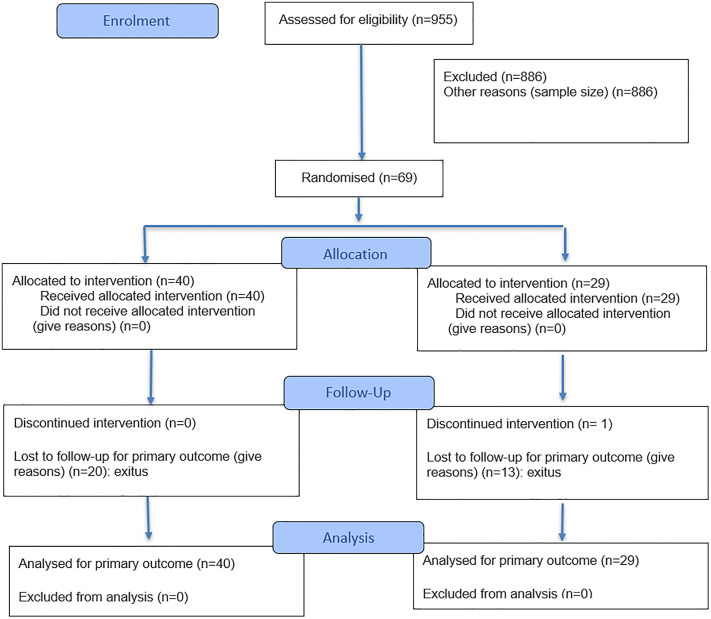
CONSORT flowchart with the development of the procedure.

Fig 1 shows the course of the procedure for both groups (January 2022 – April 2023). A total of 69 patients with a median age of 82 years were enrolled. Of the study participants, 55.1% were female and 44.9% were male.

### Clinical categories and complexity criteria in multipathologic patients

Approximately 4 out of 10 participants (42.0%; n = 29) presented a single clinical category for chronic disease, with ischemic heart disease presenting the highest percentage of patients (30.4%; n = 21). One patient presented 7 clinical categories. A complexity criterion was present in 58.0% of the participants. Polymedication (10 or more chronic prescription active ingredients) was the most prevalent criterion with 53.6% (n = 37). In addition, 33.3% (n = 23) had two or more hospital admissions in the previous 12 months (Table 1).

**Table 1:** Clinical categories and complexity criteria.

Variable	N = 69¹
**Clinical categories for chronic disease**	
Heart failure that in a situation of clinical stability has been in NYHA grade II	15 (21.7)
Ischemic heart disease	21 (30.4)
Vasculitis and systemic autoimmune diseases	1 (1.4)
Chronic renal disease defined by glomerular filtration rate <60 ml/min or albumin-creatinine index >30 mg/g	18 (26.1)
Chronic respiratory disease in a clinically stable situation that has been with grade 2 dyspnea from to MRC or FEV1 < 70% or O_2_ < 90 saturation	6 (8.7)
Inflammatory bowel disease	0 (0.0)
Chronic liver disease with data of hepatocellular insufficiency or portal hypertension	3 (4.3)
Cerebrovascular stroke	11 (15.9)
Neurological disease with permanent motor deficit causing limitation for BADL (Barthel index<60)	13 (18.8)
Neurological disease with persistent cognitive impairment, at least moderate	17 (24.6)
Symptomatic peripheral artery disease	1 (1.4)
Diabetes mellitus with proliferative retinopathy or symptomatic neuropathy	3 (4.3)
Chronic anemia due to digestive losses or acquired hemopathy not requiring curative treatment with Hb < 10 g/dL in two determinations separated by more than three months	5 (7.2)
Active solid or hematologic neoplasm not subsidiary of treatment with curative intent	8 (11.6)
Chronic osteoarticular disease that causes, by itself, a limitation for the patient to move, by himself, safely from the bed to the chair or wheelchair	5 (7.2)
Having had an osteoporotic hip fracture	4 (5.8)
**Number of clinical categories for chronic disease**	
1 clinical category	29 (42.0)
2 clinical categories	24 (34.7)
3 clinical categories	13 (18.8)
4 clinical categories	2 (2.8)
5 clinical categories	0 (0.0)
6 clinical categories	0 (0.0)
7 clinical categories	1 (1.4)
**Complexity criteria**	
Severe mental disorder (schizophrenia, manic-depressive psychosis, major depression)	6 (8.7)
Extreme polymeditation (10 or more chronically prescribed active ingredients)	37 (53.6)
Socio-familial risk (Gijon scale score greater than 10 points)	0 (0.0)
Stage II or higher-pressure ulcers	6 (8.7)
Current delirium or episodes of delirium in previous hospital admissions	0 (0.0)
Malnutrition (BMI < 18.5)	5 (7.2)
Chronic prescription tube feeding (3 or more months)	1 (1.4)
Two or more hospital admissions in the previous 12 months	23 (33.3)
Alcoholism	2 (2.9)
No complexity criteria	10 (14.5)
**Number of complexity criteria**	
No complexity criteria	10 (14.5)
1 complexity criterion	40 (58.0)
2 complexity criteria	16 (23.2)
3 complexity criteria	3 (4.4)

¹ Number of participants (frequency (%)).

NYHA: New York Heart Association; MRC: Medical Research Council; FEV1: Forced Expiratory Volume in 1 Second; O_2_: Oxygen; BADL: Basic Activities of Daily Living; Hb: Hemoglobin; BMI: Body Mass Index.

### Clinical, anthropometric and nutritional variables

Table 2 shows that the median age is higher in the Atémpero® group, although the percentage of patients with total dependence is lower (55.0% vs 72.4%). Arterial hypertension was the most prevalent previous pathology in both groups (68.9%; 67.5%), followed by diabetes mellitus (41.4%; 52.5%). Anthropometric variables did not show statistically significant differences between groups.

**Table 2:** Clinical, anthropometric and nutritional outcomes of participants.

Variable	Control Group (n = 29)^1,2^	Atémpero^®^ Group (n = 40)^1,2^	p
**Age (years)**	78 (73.0;87.0)	85 (75.5;87.2)	0.348
**Diseases**			
Diabetes Mellitus	12 (41.4)	21 (52.5)	
Arterial Hypertension	20 (68.9)	27 (67.5)	
Dyslipidemia	8 (27.6)	10 (25)	
Chronic obstructive pulmonary disease	5 (17.2)	2 (5)	0.385
Chronic Kidney Disease	4 (13.8)	8 (20)	
Congestive heart failure	4 (13.8)	8 (20)	
Neurological disease	6 (20.7)	9 (22.5)	
Oncological disease	6 (20.7)	4 (10)	
**Barthel score**			
Independent	1 (3.4)	0 (0.0)	
Mild	0 (0.0)	0 (0.0)	
Moderate	2 (6.9)	4 (10.0)	0.201
Severe	5 (17.2)	14 (35.0)	
Total	21 (72.4)	22 (55.0)	
**Current weight(kg)**	64.4 ± 13.8	64.7 ± 14.8	0.919
**Usual weight(kg)**	73.4 ± 13.6	72.2 ± 14.2	0.756
**Weight loss**	35 (87.5)	25 (86.2)	0.943
**% weight loss**	8.4 (4.7;13.9)	8.0 (4.2;16.2)	0.923
**Height (m)**	1.64 ± 0.1	1.63 ± 0.1	0.768
**BMI (kg/m**^**2**^)	24.3 ± 6.7	24.5 ± 6.0	0.904
**BMI Category**			
Underweight	3 (10.3)	7 (17.5)	
Normal weight	15 (51.7)	17 (42.5)	
Overweight grade I	2 (6.9)	6 (15.0)	
Overweight grade II	6 (20.7)	2 (5.0)	0.161
Obesity type I	1 (3.4)	6 (15.0)	
Obesity type II	1 (3.4)	2 (5.0)	
Obesity type III	1 (3.4)	0 (0.0)	
**Tricipital skin fold (mm)**	10.0 (6.0;14.0)	11.0 (8.0;20.0)	0.079
**Subscapular skin fold (mm)**	11.0 (8.0;19.7)	13.0 (9.5;18.0)	0.303
**Calf circumference (cm)**	29 (25.9;31.0)	29.5 (28.0;32.0)	0.202
**Arm circumference (cm)**	26.5 (24.0;31.0)	28.0 (25.2;29.9)	0.316
**Bioimpedanciometry**			
Phase angle	4.0 (3.3;4.5)	3.9 (3.5;4.7)	0.839
FM (kg)	12.8 (7.2;17.6)	12.0 (7.5;16.2)	0.802
<mean	4 (13.8)	5 (12.5)	
= mean	9 (31.0)	13 (32.5)	0.803
>mean	15 (51.7)	18 (45.0)	
FFM (kg)	26.9 (24.4;29.3)	26.7 (25.0;29.4)	0.855
<mean	12 (41.4)	5 (12.5)	
= mean	12 (41.4)	26 (65.0)	>0.05^3^
>mean	4 (13.8)	5 (12.5)	
BCM (kg)	10.7 (9.0;12.2)	11.3 (9.6;12.4)	0.389
<mean	20 (68.9)	19 (47.5)	
= mean	6 (20.7)	15 (37.5)	0.333
>mean	2 (6.9)	2 (5.0)	
SMI (kg)	8.0 (6.6;8.5)	7.5 (6.1;8.7)	0.876
<mean	17 (58.6)	20 (50.0)	
= mean	1 (3.4)	2 (5.0)	0.782
>mean	10 (34.5)	14 (50.0)	
% Hydration	73.8 (73.2;81.6)	74.1 (73.5;81.1)	0.583
<mean	4 (13.8)	2 (5.0)	
= mean	13 (44.8)	19 (47.5)	0.536
>mean	11 (37.9)	15 (37.5)	
TBW (L)	27.0 (18.1;23.4)	20.9 (18.6;24.3)	0.516
<mean	3 (10−3)	2 (5.0)	
= mean	17 (58.6)	21 (52.5)	0.622
>mean	8 (27.6)	13 (32.5)	
**MNA score**			
Risk of malnutrition	2 (6.9)	8 (20.0)	0.174
Malnutrition	27 (93.1)	32 (80.0)	
**GLIM phenotypic criteria**			
Unintentional weight loss	24 (82.8)	31 (77.5)	
Low BMI	11 (37.9)	12 (30.0)	0.922
Reduced muscle mass	22 (75.9)	35 (87.5)	
**GLIM etiologic criteria**			
Reduced food intake or absorption	24 (82.8)	35 (87.5)	0.426
Inflammation	25 (86.2)	33 (82.5)	
**GLIM etiological criteria**			
Moderate malnutrition	21 (72.4)	32 (80.0)	0.654
Severe malnutrition	8 (27.6)	8 (20.0)	
**% of intake**			
0% intake	2 (6.9)	4 (10.0)	
25% intake	13 (44.8)	13 (32.5)	
50% intake	8 (27.6)	13 (32.5)	0.690
75% intake	2 (6.9)	1 (2.5)	
100% intake	4 (13.8)	9 (22.5)	
**Previous EN**	9 (31.0)	12 (30.0)	0.926

^1^Number of participants (frequency (%)); ^2^ Median (interquartile range); ^3^After Benjamini-Hochberg procedure (False Discovery Rate). BMI: Body Mass Index; FM: Fat Mass; FFM: Fat-Free Mass; BCM: Body Cell Mass; SMI: Muscle Mass Index; TBW: Total Body Water; MNA: Mini-Nutritional Assessment; GLIM: Global Leadership Initiative on Malnutrition; EN: Enteral Nutrition

**Fig 2:**
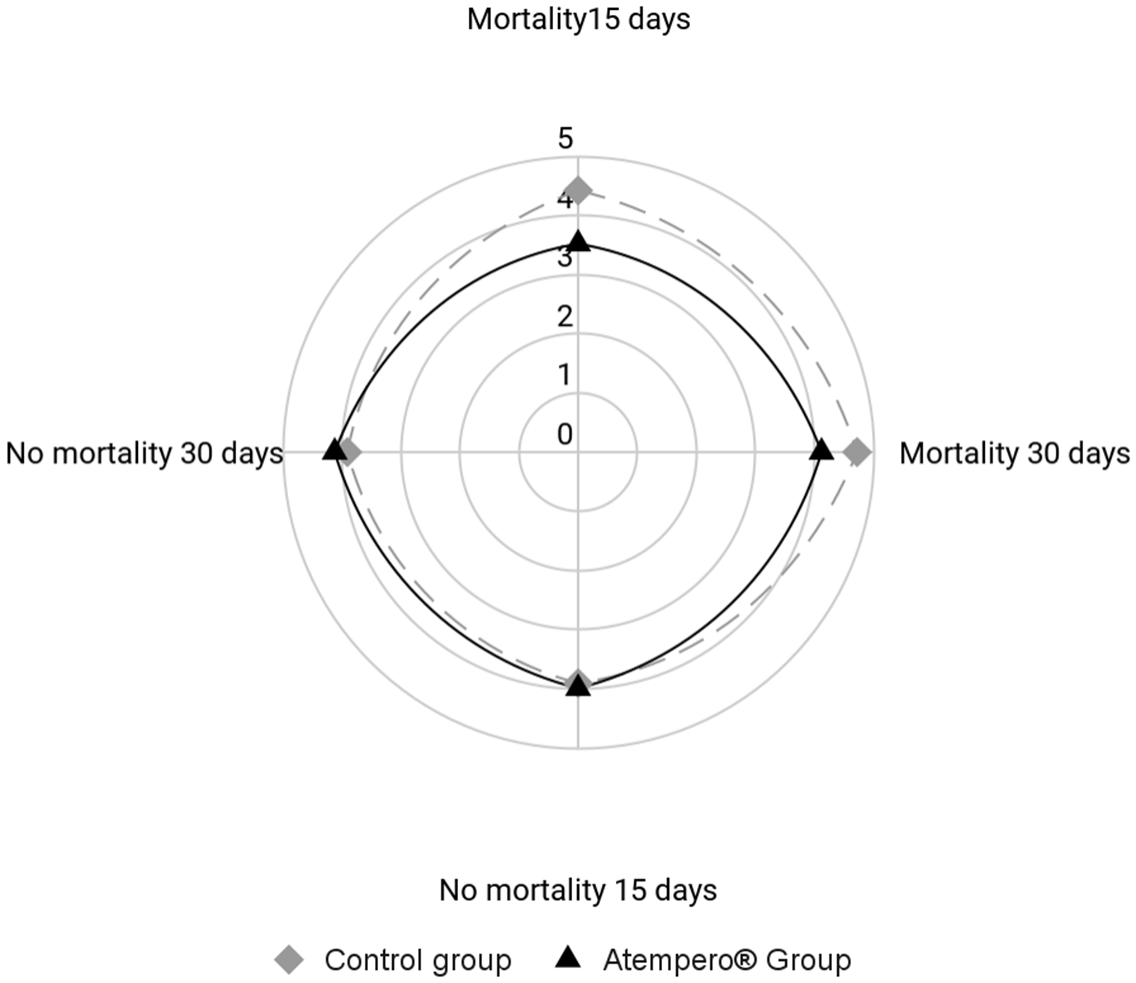
Phase angle and mortality.

After 15 days, 7 participants in the control group and 14 patients in the Atémpero^®^ group died (1 participant in the Atémpero^®^ group revoked consent and left the study). Taking the phase angle value in the initial collection, patients who died in the first 15 days had a lower PA value than the initial one (4.4º vs 4.7º in the control group; 3.5º vs 4.1º in the Atémpero^®^ group). The FA value for patients who completed the study was lower than or maintained the FA at 15 days (3.9º in the control group; 4.1º in the Atémpero^®^ group) (Fig 2).

Table 3 shows the changes in the degree of dependence, weight, weight loss and dietary intake during the study period, adding adherence to the prescribed ONS. There were no statistically significant differences between the groups. There was an increase in participants who ingested 100% of the intake (36.4% vs 50.0% in the control group; 34.6% vs 54.4% in the Atémpero® group). Adherence to the ONS improved at visit 3 but did not reach 100% of the study population.

**Table 3:** Evolution of clinical, anthropometric and nutritional variables.

	15 days			30 days		
Variable	Control Group (n = 22)^1,2^	Atémpero® Group (n = 26)^1,2^	p	Control Group (n = 20)^1,2^	Atémpero® Group (n = 22)^1,2^	p
**Barthel score**			0.209			0.272
Independent	1 (4.5)	0 (0.0)		1 (5.0)	0 (0.0)	
Mild	1 (4.5)	0 (0.0)		3 (15.0)	2 (9.1)	
Moderate	2 (9.1)	8 (30.8)		3 (15.0)	5 (22.7)	
Severe	4 (18.2)	6 (23.1)		0 (0.0)	4 (18.2)	
Total	14 (63.6)	12 (46.1)		13 (65.0)	11 (50.0)	
**Weight (kg)**	68.0 (61.0;77.2)	62.6 (54.0;72.8)	0.288	67.6 (63.0;73.6)	61.5 (54.2;69.4)	0.503
**Weight loss**	5 (22.7)	5 (19.2)	0.408	6 (30.0)	5 (22.7)	0.552
**% of intake**			0.196			0.823
0% intake	0 (0.0)	1 (3.8)		0 (0.0)	1 (4.5)	
25% intake	1 (4.5)	4 (15.4)		3 (15.0)	2 (9.1)	
50% intake	2 (9.1)	6 (23.1)		3 (15.0)	4 (18.2)	
75% intake	11 (50.0)	6 (23.1)		3 (15.0)	3 (13.6)	
100% intake	8 (36.4)	9 (34.6)		10 (50.0)	12 (54.5)	
**ONS adherence**			0.694			0.107
0% intake	1 (4.5)	1 (3.8)		1 (5.0)	2 (9.1)	
25% intake	2 (9.1)	1 (3.8)		2 (10.0)	1 (4.5)	
50% intake	3 (13.6)	4 (15.4)		4 (20.0)	5 (22.7)	
75% intake	2 (9.1)	3 (11.5)		4 (20.0)	0 (0.0)	
100% intake	13 (59.1)	17 (65.4)		7 (35.0)	14 (63.6)	
Not available	1 (4.5)	0 (0.0)		2 (10.0)	0 (0.0)	

^1^Number of participants (frequency (%)); ^2^ Median (interquartile range).

ONS: Oral Nutritional Supplements.

#### Risk and presence of PRI.

Table 4 shows that the presence of PRI in the Atémpero® group was higher than in the control group (47.5% vs 37.9%). In addition, they presented a higher prevalence of high risk of presenting PRI according to the Braden scale (72.5% vs 68.9%). They also obtained a higher value on the PUSH scale (12.0 vs 11.0), mainly due to the values according to the size of PRI and the amount of exudate.

**Table 4:** Risk and presence of initial PRI by group.

Variable	Control Group (n = 29)^1,2^	Atémpero^®^ Group (n = 40)^1,2^	p
**Braden score**			0.861
Low risk	1 (3.4)	2 (5.0)	
Moderate risk	8 (27.6)	9 (22.5)	
High risk	20 (68.9)	29 (72.5)	
**Presence of PRI**	11 (37.9)	19 (47.5)	0.585
**PRI categorization**			0.161
Category I	4 (36.3)	3 (15.8)	
Category II	2 (18.2)	6 (31.6)	
Category III	3 (27.3)	10 (52.6)	
Category IV	2 (18.2)	0 (0.0)	
**PRI size, PUSH scale (cm**^**2**^)			0.483
<0.3	0 (0.0)	0 (0.0)	
0-3-0.6	1 (9.1)	1 (5.2)	
0.7-1.0	0 (0.0)	2 (10.5)	
1.1-2.0	0 (0.0)	0 (0.0)	
2.1-3.0	0 (0.0)	0 (0.0)	
3.1-4.0	1 (9.1)	0 (0.0)	
4.1-8.0	2 (18.2)	3 (15.8)	
8.1-12.0	1 (9.1)	4 (21.0)	
12.1-24.0	3 (27.3)	2 (10.5)	
>24.0	2 (18.2)	7 (36.8)	
**Amount of PRI exudate, PUSH scale**			0.708
None	6 (54.5)	6 (31.6)	
Slight	2 (18.2)	6 (31.6)	
Moderate	1 (9.1)	2 (10.5)	
Abundant	2 (18.2)	5 (26.3)	
**PRI tissue type, PUSH scale**			0.480
Closed	2 (18.2)	1 (5.2)	
Epithelial tissue	2 (18.2)	3 (15.8)	
Granulation tissue	1 (9.1)	4 (21.0)	
Sphacelium	1 (9.1)	6 (31.6)	
Necrotic tissue	5 (45.4)	5 (26.3)	
**Total PUSH scale score**	11 (6.0;13.0)	12.0 (8.5;15.5)	0.353

^1^Number of participants (frequency (%)); ^2^Median (interquartile range).

PRI: Pressure-related injury; PUSH: Pressure Ulcer Scale for Healing.

**Fig 3:**
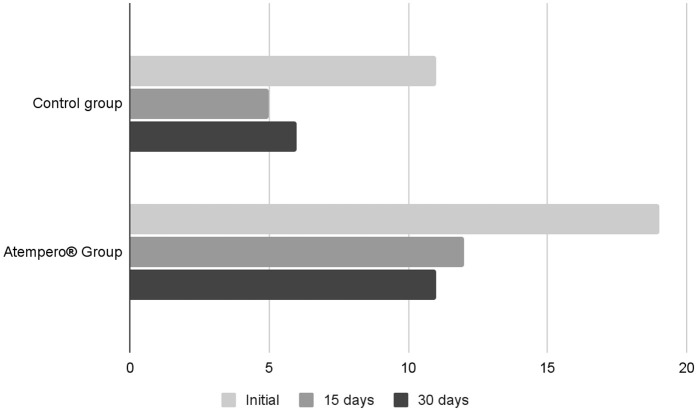
Evolution of the presence of PRI per group.

A descriptive reduction in the absolute number of patients with PRIs was observed at 15 and 30 days within the Atémpero^®^ group, whereas an increase was noted in the control group; however, both groups exhibited improvements by the study’s conclusion (n = 11 vs n = 5 vs n = 6 in the control group and n = 19 vs n = 12 vs n = 11 in the Atémpero^®^ group) (Fig 3).

**Fig 4:**
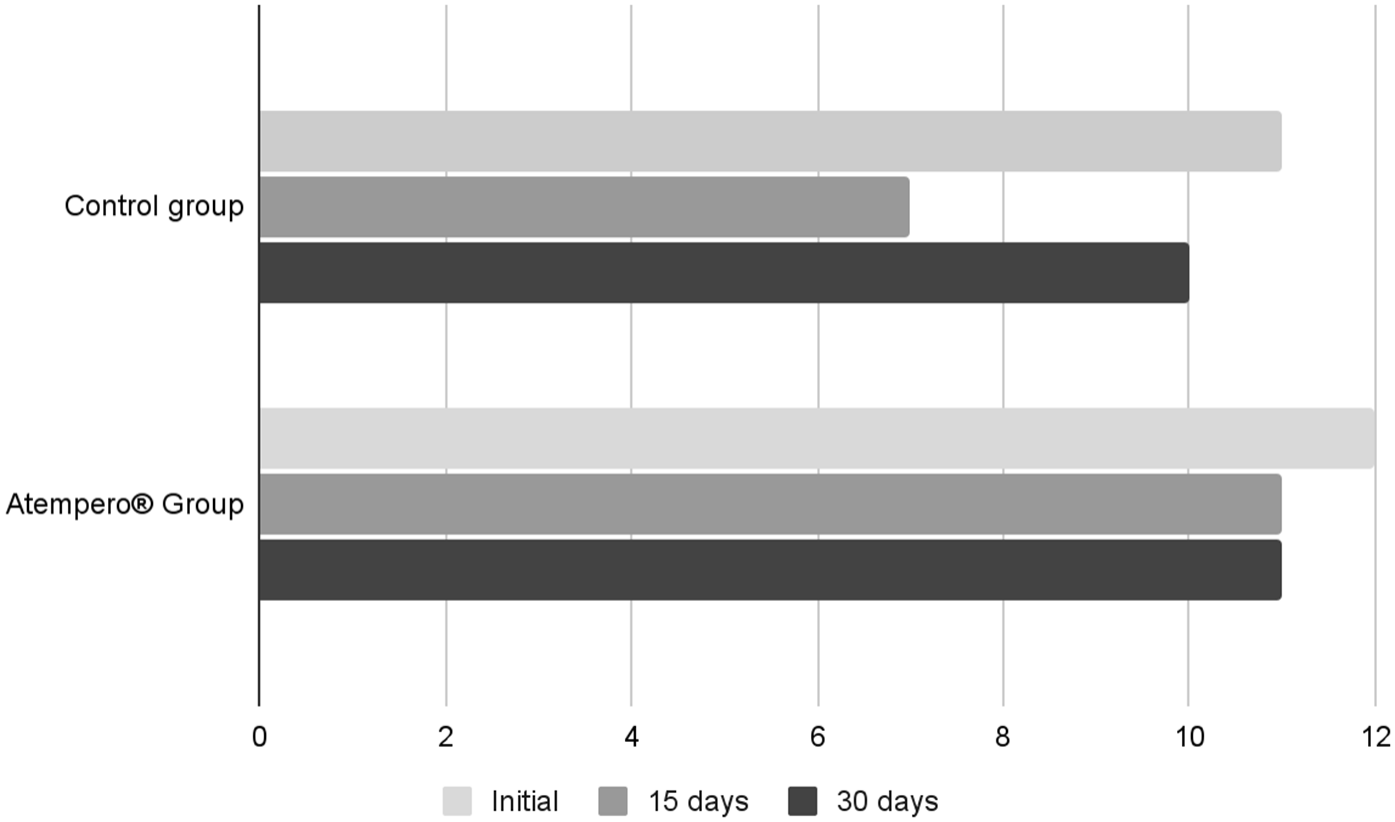
Evolution of the PUSH scale score per group.

Another aspect studied was the score on the PUSH scale in those patients who presented PRI, which decreased at 15 days and increased or remained stable at 30 days, depending on the group under study (Fig 4).

Table 5 shows how the high risk of presenting a PRI, according to the Braden scale, decreased in both groups, with a greater decrease in the Atémpero® group (72.5% vs 40.9%), as well as the categorization and the parameters that form part of the PUSH scale.

**Table 5:** PUSH scale risk, categorization and parameters.

	15 days			30 days		
	Control Group (n = 22)^1,2^	Atémpero^®^ Group (n = 26)^1,2^	p	Control Group (n = 20)^1,2^	Atémpero^®^ Group (n = 22)^1,2^	p
**Braden scale result (risk)**			0.607			0.688
Low	3 (13.6)	4 (15.4)		5 (25.0)	6 (27.3)	
Moderate	5 (22.7)	9 (34.6)		4 (20.0)	7 (31.8)	
High	14 (63.6)	13 (50.0)		10 (50.0)	9 (40.9)	
**PRI categorization**			0.255			0.428
Category I	1 (20.0)	3 (25.0)		2 (33.3)	2 (18.2)	
Category II	2 (40.0)	4 (33.3)		1 (16.6)	5 (45.4)	
Category III	1 (20.0)	5 (41.6)		3 (50.0)	4 (36.3)	
Category IV	1 (20.0)	0 (0.0)		0 (0.0)	0 (0.0)	
**PRI size, PUSH scale (cm**^**2**^)			0.410			0.275
<0.3	0 (0.0)	1 (8.3)		1 (16.6)	0 (0.0)	
0-3-0.6	1 (20.0)	1 (8.3)		1 (16.6)	0 (0.0)	
						
0.7-1.0	1 (20.0)	1 (8.3)		0 (0.0)	1 (9.1)	
1.1-2.0	1 (20.0)	0 (0.0)		0 (0.0)	1 (9.1)	
2.1-3.0	0 (0.0)	0 (0.0)		0 (0.0)	0 (0.0)	
3.1-4.0	1 (20.0)	1 (8.3)		1 (16.6)	0 (0.0)	
4.1-8.0	0 (0.0)	0 (0.0)		1 (16.6)	0 (0.0)	
8.1-12.0	1 (20.0)	2 (16.6)		1 (16.6)	5 (45.4)	
12.1-24.0	0 (0.0)	4 (33.3)		1 (16.6)	2 (18.2)	
>24.0	0 (0.0)	2 (16.6)		0 (0.0)	2 (18.2)	
**Amount of PRI exudate, PUSH scale**			0.181			0.520
None	3 (60.0)	5 (41.6)		3 (50.0)	5 (45.4)	
Slight	0 (0.0)	4 (33.3)		1 (16.6)	4 (36.6)	
Moderate	2 (40.0)	2 (16.6)		2 (33.3)	2 (18.2)	
Abundant	0 (0.0)	1 (8.3)		0 (0.0)	0 (0.0)	
**PRI tissue type, PUSH scale**			0.220			0.308
Closed	1 (20.0)	1 (8.3)		1 (16.6)	1 (9.1)	
Epithelial tissue	0 (0.0)	3 (25.0)		1 (16.6)	2 (18.2)	
Granulation tissue	0 (0.0)	4 (33.3)		1 (16.6)	3 (27.3)	
Sphacelium	3 (60.0)	3 (25.0)		1 (16.6)	5 (45.4)	
Necrotic tissue	1 (20.0)	1 (8.3)		2 (33.3)	0 (0.0)	

^1^Number of participants (frequency (%)); ^2^ Median (interquartile range).

PRI: Pressure-related injury; PUSH: Pressure Ulcer Scale for Healing.

## Discussion

The prevention and management of PRIs play an essential role in health care, especially in elderly patients with multiple pathologies [27]. Age, chronic diseases, polypharmacy [28] and dependence [29] increase the risk of presenting PRI directly or indirectly through the reduction of food intake [30]. Our results show that polypharmacy is very present since 53.6% of the participants were prescribed ten or more active ingredients. The degree of total dependence in the initial population, according to the Barthel scale, was present in 6 out of 10 participants in the sample (62.3%), being higher in the control group (72.4%). This situation implies an increase in morbidity and hospital stays, which causes a high economic burden for the healthcare systems [31]. Of the participants, 33.3% had two or more admissions in the previous 12 months.

As previously mentioned, these patients’ food intake should be assessed [32]. 18% of the study participants ingested the entire prescribed diet, and 46.4% ingested less than 50% of the diet. Intake improved during the process, with the improvement in intake being similar in both groups (36.4% at 15 days and 50.0% at 30 days vs 34.6% and 54.6%). These data are opposed by Wong et al., who concluded that the prescription of an oral fortified enteral formula vs a standard diet did not modify nutritional intake [33] since there was an improvement in both groups during the study interval.

Nutritional screening and correct diagnosis of malnutrition are essential since, as mentioned, the risk of malnutrition or malnutrition already in place plays a fundamental role in skin integrity [34,35]. Body composition at the beginning of the study was similar in both groups. However, there were statistically significant differences (p = 0.038) in fat-free mass, taking the average values for each age as a reference. The control group presented a higher percentage of patients below the normal range (41.4% vs 12.5%). In both groups, almost 9 out of 10 participants had presented weight loss in the previous six months. Both ONSs formulas decreased weight loss at 15 days (22.7% control group; 19.2% Atémpero® group) with an increase in patients with weight loss at 30 days (30.0% control group; 22.7% Atémpero® group), although this was lower than that presented at the beginning of the study.

Of the sample, 100.0% were moderately or severely malnourished, although only 7.2% of the patients were classified as malnourished within the complexity criteria. The nutritional management of patients at risk or with PRI is the most influential factor for its prevention and healing [36]. Within this management is the use of enteral nutrition if the patient cannot meet his nutritional requirements. Our results showed that only 3 out of 10 participants received some EN, and none received individualized nutritional counselling. The lack of oral nutritional supplementation in patients with low intake increases the risk of developing PRI [37].

According to the data obtained, the high risk of developing PRI according to the Braden scale was higher in the Atémpero® group (72.5% vs 68.9%). The 91.3% presented a moderate or high risk of presenting PRI. This risk decreased during the study in both groups. 43.5% presented PRI at the start of the study, being higher in the Atémpero® group (19 = 47.5%; 11 = 37.9%). The presence of PRI in absolute numbers (number of patients) was reduced at 15 and 30 days in the Atémpero® group and increased in the control group, although both improved at the end of the study (n = 11 vs n = 6 in the control group and n = 19 vs n = 11 in the Atémpero® group). The categorization of PRIs in the Atémpero® group improved by decreasing the percentages in higher categories. These data coincide with those reported by other researchers, such as Cereda et al., where they obtained that patient who received a hyperproteic oral formula enriched with arginine, zinc and vitamin C presented a higher cure rate [2,38]. This conclusion was also reinforced by Bauer et al., concluding that using nutritional supplements enriched with nutrients improves healing in relation to using a standard formula [39]. Other authors concluded that using nutrients such as arginine in Atémpero® improves wound healing [22,40].

**Fig 5:**
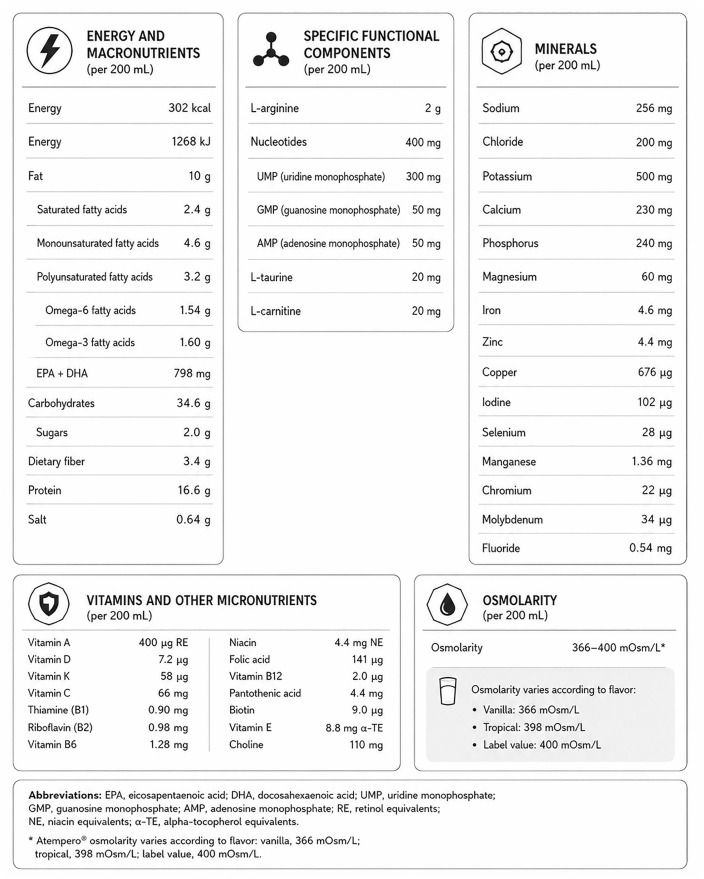
Nutrient profile of Atémpero formula.

Scores on the PUSH scale decreased at 15 days and increased or remained stable at 30 days depending on the study group (control 11 vs 7 vs 10 and Atémpero® 12 vs 11 vs 11). These data show that the use of a nutrient-enriched hypercaloric and hyperprotein formula (Fig 5) reduced PUSH scores to a lesser extent than a standard hyperprotein and hypercaloric formula at 15 days, but showed descriptively lower PUSH scores at 30 days. Among the parameters studied for obtaining the PUSH score was the area of the PRI. Cereda et al. found that participants taking a hypercaloric and hyperprotein diet enriched with immunonutrients reduced the PRI area after eight weeks (60.9% vs 45.2%) [41]. After four weeks of supplementation, the PRI area was reduced in both groups.

Although statistical significance was not reached—potentially due to the limited sample size—the observed reduction in PUSH scores and the downward shift in Braden risk categories within the Atémpero^®^ group represent descriptive trends that warrant further evaluation in larger cohorts. In geriatric, multi-pathological populations, even incremental improvements in skin integrity and risk reduction can significantly decrease the probability of infection and associated mortality, which are critical outcomes for patient safety [42–44].

These findings align with the principles outlined by various national and international organizations regarding nutritional support. According to the NICE (National Institute for Health and Care Excellence) and Wound Healing Society guidelines, the prescription of nutritional supplementation is only recommended when the patient has an inadequate intake or in the presence of a nutritional deficit [45]. The ESPEN guideline recommends (grade B) offering nutritional interventions to older patients at risk of PRI to prevent development. Nutritional interventions should be offered to malnourished older people with PRI to improve healing [18]. Although the findings lack statistical significance, the observed trends align with the clinical principles outlined by the National Group for the Study and Advice on Pressure Ulcers and Chronic Wounds (GNEAUPP) in Spain, published in Technical Document No. XII, which states that the consumption of supplements that provide vitamins A, C, E, zinc and arginine improves the prevention (high level of evidence) and healing of PRI (moderate level of evidence) [1]. Overall, future studies should also analyse biomarkers related to immunity in this type of oral supplementation.

The study has limitations that make it difficult to generalize its results, but it should be noted that the results obtained are a starting point for future, more specific research. One limitation is the insufficient sample size due to the difficulty in recruiting participants given their clinical characteristics and the setting in which the study was conducted, which may lead to a risk of type II error. Although comparatively, it presents more participants than similar studies [37–39]. The mean age between the two groups differs, with the Atémpero® group having a higher mean age, which suggests a higher degree of baseline frailty in this group. Another limitation is the high mortality rate of our participants, which could be a consequence of the diseases present, malnutrition and the presence of PRI, all of which aggravate morbidity and mortality [46]. Our data show that 6 participants died in the control group, of whom 4 (66.6%) had PRI. In the Atémpero® group, 14 patients died, 7 of whom had PRI (50.0%). At 30 days, 2 participants died in the control group, 0 (0.0%) of whom had pre-PRI. In the Atémpero® group, there were 4, of which 1 had PRI (25.0%). Future studies should take special care to recruit a sufficient number of participants to ensure that the analysis can be carried out, taking into account the losses that may arise from the process due to the clinical conditions of this type of participant. In addition, adherence to the ONS was low since not all patients took 100% of the regimen due to clinical conditions, although most took 50% or more. Another possible factor hindering significant results is the clinical approach to wounds and the interprofessional variability in this approach. Another aspect to be taken into account is that all the malnourished patients were prescribed ONS on an individualized basis, which makes it difficult to compare with other studies in which the control group did not take ONS or took a normoprotein and isocaloric formula or did not undergo a prior nutritional assessment for a correct diagnosis of malnutrition.

## Conclusions

Patients with multiple pathologies present factors that increase the risk of presenting PRI: chronic diseases, polypharmacy, dependence and alterations in nutritional status. Therefore, carrying out a correct nutritional assessment and early intervention is crucial. While the administration of the intervention ONS was associated with descriptive variations in PRI risk and healing, the lack of statistical significance requires these results to be interpreted strictly as exploratory trends. Due to the lack of statistical significance, these findings provide a basis for future confirmatory trials with larger sample sizes to validate the definitive efficacy of this specific immunonutrient profile in multi-pathological populations.

## Supporting information

S1 Text CONSORT 2025 checklist. (PDF)
